# Mercury: Cleanup for Broken CFLs

**DOI:** 10.1289/ehp.116-a378

**Published:** 2008-09

**Authors:** Graeme Stemp-Morlock

Compact fluorescent lamps (CFLs) are about 75% more energy efficient than incandescent light bulbs and last 10 times longer, and thus have quickly become a modern-day environmental icon. The U.S. Environmental Protection Agency (EPA) estimates that about 290 million CFLs were sold in 2007. But CFLs do have one dim spot on their otherwise bright green image: the mercury that makes the bulbs’ inner phosphor coating fluoresce and produce light. A new study from a group of researchers at Brown University characterizes for the first time how elemental mercury vapor escapes from broken CFLs and offers a real-world solution for capturing escaping mercury.

According to a June 2008 fact sheet issued by the EPA Energy Star program, the use of CFLs results in a net reduction in mercury entering the environment because their lower energy draw means less mercury-emitting coal needs to be burned. The EPA estimates that using a 13-W CFL saves 376 kWh over its 8,000-hour lifespan, preventing 4.5 mg of mercury from being emitted by a coal-burning power plant. Each small, curly tube contains about 3–5 mg of mercury—significantly less than the 500 mg in older thermometers, but enough that environmental and human health concerns remain.

The research group headed by Robert Hurt, director of the Institute for Molecular and Nanoscale Innovation, broke a series of new and used CFLs to measure the release of mercury vapor into the air. In the hour immediately after each breakage, the team recorded mercury gas concentrations near the bulb shards between 200–800 μg/m^3^. For comparison, the average 8-hour occupational exposure limit allowed by the Occupational Safety and Health Administration is 100 μg/m^3^. Within 4 days a new 13-W CFL released about 30% of its mercury, with the remainder appearing to remain trapped in the bulb debris; picking up the glass shards after breakage reduced mercury release by 67%. Used bulbs followed similar patterns but with lower rates. The study, which was funded by the NIEHS Superfund Basic Research Program, was reported in the 1 August 2008 issue of *Environmental Science & Technology*.

“The amount of mercury gas coming off [broken CFLs] is over a milligram over a few days. If you put that milligram into a poorly ventilated room, the concentration can be over the recommended limit for children [of 0.2 μg/m^3^],” says Hurt. “The overall risk is low, but it’s not zero risk, and there is definitely an opportunity to do better.”

This kind of information could help regulators provide better information on how to handle broken CFLs. In 2007 the Maine Department of Environmental Protection performed one of the only other studies evaluating mercury exposure from broken CFLs. The EPA’s current recommendation to leave the room for at least 15 minutes immediately after breaking a CFL derives from that study. The EPA also recommends that broken CFL pieces be scooped up and placed in a plastic bag.

However, Hurt’s research suggests that the peak for escaping mercury vapor lasts a few hours. The group also found that plastic bags leaked mercury vapor. “This new information may allow for modeling of airborne mercury concentrations following breakage, thus providing the capability to more fully assess the effectiveness of cleanup,” says Roxanne Smith, a press officer for the EPA.

Hurt’s group also tested 28 sorbents for their ability to capture the released mercury gas. Because a sorbent’s surface area can affect how well it captures mercury, the team chose to test nanoscale formulations, which provide large surface area. One type of nanoselenium was found to be the most effective, removing 99% of the mercury vapor when impregnated in a cloth that was draped over a broken CFL or sprinkled over the breakage as a powder. When the mercury vapor reacted with nanoselenium, it formed mercury selenides, which are insoluble and metabolically inactive, according to a report in the November 2004 issue of the *Seychelles Medical and Dental Journal*. These compounds are also believed to be stable under landfill conditions (with the caveat that the environmental disposition and health effects of nanomaterials are still largely unknown).

There are CFL recycling programs across the country at major retailers such as The Home Depot, but the Association of Lighting and Mercury Recyclers estimates that 98% of CFLs currently end up in landfills. Hurt’s group has therefore developed prototype packaging and disposal bags that can act as a barrier to prevent mercury from escaping as well as neutralize it. “Development of technology or material to more effectively clean up or capture mercury vapor may potentially minimize worker exposures during transport and disposal and, if readily available to consumers, may potentially minimize future inhalation exposures in residential settings,” says Smith.

## Figures and Tables

**Figure:**
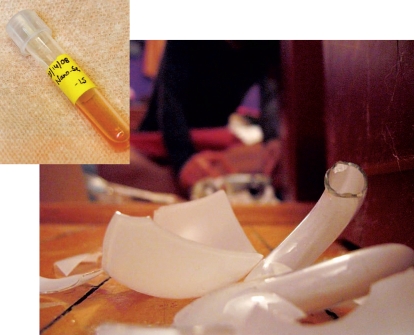
Nanoselenium (inset) may be one answer to the question of how to safely clean up mercury from broken CFLs

